# SLID: a slit-lamp image dataset for deep learning-based anterior eye anatomical segmentation and multi-lesion detection

**DOI:** 10.3389/fdgth.2025.1716501

**Published:** 2026-01-12

**Authors:** Mingyu Xu, Yiming Sun, Huimin Cheng, Yifan Zhou, Nuliqiman Maimaiti, Pengjie Chen, Qi Miao, Peifang Xu, Juan Ye

**Affiliations:** 1 Eye Center of Second Affiliated Hospital, School of Medicine, Zhejiang University, Hangzhou, Zhejiang, China; 2 Zhejiang Provincial Key Laboratory of Ophthalmology, Zhejiang Provincial Clinical Research Center for Eye Diseases, Zhejiang Provincial Engineering Institute on Eye Diseases, Hangzhou, Zhejiang, China; 3 School of Software Technology, Zhejiang University, Hangzhou, Zhejiang, China

**Keywords:** anatomical segmentation, dataset, deep learning, lesion detection, ocular anterior segment disease, open source

## 1 Introduction

Ocular anterior segment diseases are a significant global public health concern, being major contributors to blindness and visual impairment worldwide (1–4). Cataract, known as the leading cause of reversible blindness and visual impairment globally (5–7), along with corneal diseases, which rank as the fourth leading cause of blindness (8, 9), underscore the urgent need for timely diagnosis and intervention, particularly for conditions like keratitis that can progress rapidly (10, 11). Among the essential tools for diagnosing and managing these conditions is the slit-lamp biomicroscope, a fundamental instrument in ophthalmology clinics known for its convenience, availability, cost-effectiveness, and efficiency in identifying common ocular anterior segment diseases.

Recent advancements in artificial intelligence (AI) have shown promising results in automated diagnosis and treatment planning based on slit-lamp images (12–23). However, these AI systems are often limited by the availability of comprehensive datasets for training and validation purposes. Currently, there is a scarcity of open-access datasets that include slit-lamp images with detailed anatomical annotations and lesion identification necessary for developing robust AI models applicable to real-world clinical scenarios.

The publication of slit-lamp image datasets with thorough anatomical annotations and lesion identification is therefore pivotal. These datasets not only facilitate the advancement of AI models capable of precise and clinically relevant diagnoses but also serve to validate existing AI technologies. By offering standardized datasets to researchers globally, we aim to expedite progress toward more efficient computer-aided diagnosis and treatment systems for ocular anterior segment diseases.

**Figure 1:**
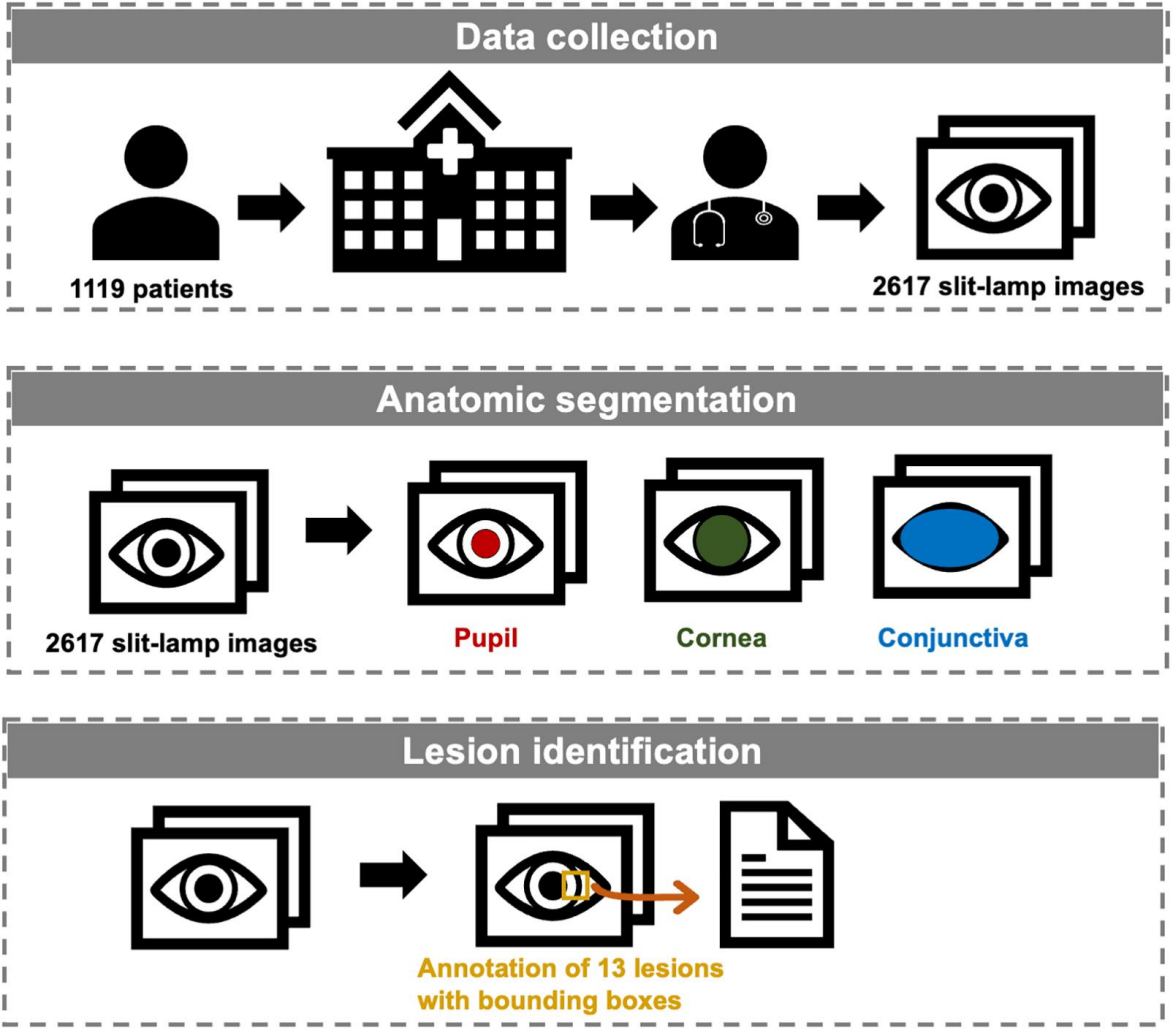
Workflow of the establishment of the slit-lamp dataset.

This paper introduces a comprehensive dataset crafted to fill these critical gaps in the field. Figure 1 illustrates our study, delineating the processes of data collection, anatomical segmentation, and lesion identification. To the best of our knowledge, this is the first open-source slit-lamp dataset with detailed anatomical and lesion-level information. Our dataset endeavors to serve as an invaluable resource for researchers endeavoring to develop AI models that are not only accurate but also applicable in preclinical screening and clinical practice.

## 2 Materials and methods

### 2.1 Patient selection and image acquisition

We retrospectively collected 2,617 ocular surface slit-lamp images from 1,119 patients between November 2016 and March 2022 at the Eye Center of the Second Affiliated Hospital of Zhejiang University, College of Medicine, China. All images were captured using Topcon SL-D701 slit-lamp biomicroscopes equipped with DC-4 digital cameras. Images were excluded based on the following criteria: (1) Poor quality images, defined as images with significant defocus or inappropriate illumination (overexposed or underexposed conditions) such that lesion features cannot be clearly identified; (2) Indeterminate lesions, defined as cases with atypical or ambiguous presentations in which our annotation team could not reach a consensus on the specific lesion category; (3) Anatomical abnormalities, defined as structural alterations of the ocular surface caused by prior surgery or trauma. To ensure anonymity, patient information was obscured with a black rectangular box in the top left corner of each image. Due to multiple follow-up visits for the same patient and varying eye positions and focus points during the same visit, multiple images may exist for the same eye of a single patient.

Demographically, a total of 1,383 eyes were included, comprising 668 left eyes and 715 right eyes, corresponding to 1,119 patients. Of these, 621 were women and 498 were men. Age distribution was as follows: 236 patients <18 years, 408 aged 18–44 years, 200 aged 45–59 years, and 275 aged ≥60 years. The number of images, eyes, and patients corresponding to each disease category was provided in Table 1.

**Table 1:** The number of images, eyes, and patients corresponding to each disease category of the SLID dataset.

Category		No. images	No. eyes	No. patients
Normal		245	220	150
Monomorbidity	Cataract	107	92	77
	Intraocular lens	69	34	31
	Lens dislocation	31	16	10
	Keratitis	162	38	37
	Corneal scarring	49	25	24
	Corneal dystrophy	289	169	108
	Corneal/conjunctival tumor	447	164	162
	Pinguecula	203	129	103
	Pterygium	92	63	51
	Subconjunctival hemorrhage	134	69	66
	Conjunctival injection	36	26	23
	Conjunctival cyst	90	43	42
	Pigmented nevus	382	175	165
Multimorbidity		281	192	179

This study received approval from the Ethics Committee of the Second Affiliated Hospital of Zhejiang University, College of Medicine (IR2021001176). All procedures conformed to the principles of the Declaration of Helsinki. Data was retrospectively collected from patients through routine medical care and obtained from medical facilities. The dataset does not include direct identifiers, as all participants’ names were removed, and their IDs were restructured to anonymize identifying information. Thus, the Ethics Committee of the Second Affiliated Hospital of Zhejiang University, College of Medicine granted a waiver of consent.

### 2.2 Annotation

The annotation data includes localization and categorical information pertaining to anatomical regions and lesions. All images underwent manual annotation using the VGG annotator by a professional team (24). This team comprised one junior ophthalmologists (JO) with over three years of clinical experience, one senior ophthalmologists (SO) with more than six years of clinical experience, and one specialized ophthalmologist with over 10 years of clinical experience. The JO and SO independently labeled the images, which were then reviewed and confirmed by the specialized ophthalmologist, and respective metadata files were exported.

For anatomical segmentation, three regions were annotated: pupil, cornea, and conjunctiva. Each image's anatomical annotations were meticulously delineated with respect to their labels. Typically, the anatomic regions of pupil and cornea were within the labeled circle and ellipse up to the conjunctival boundary (if intersected). In cases where the exposed areas of the pupil or cornea were too small to be labeled by circle or ellipse, polygonal contours were used. The conjunctiva was consistently contoured using polygons across all cases. It is important to note that certain anatomical regions may not be labeled if they are excluded from the image or if their edges cannot be determined due to the presence of lesions. An example of anatomical annotation is shown in Supplementary Figure S1.

For lesion identification, we identified 13 common classes encountered in clinical practice: cataract, intraocular lens, lens dislocation, keratitis, corneal scarring, corneal dystrophy, pinguecula, pterygium, subconjunctival hemorrhage, conjunctival injection, conjunctival cyst, pigmented nevus, and corneal/conjunctival tumor. Lesions were annotated using bounding boxes, except for cataract, intraocular lens, lens dislocation, and conjunctival injection. These lesions encompass the entire pupil or conjunctival area, so they were localized as the respective anatomical region. For normal images without detected lesions, only anatomical annotations were included. Examples illustrating lesion annotations for all monomorbidity cases and an example of multimorbidity are depicted in Supplementary Figure S2.

### 2.3 Data description

The dataset is available on the Github, with a summary of its features provided in Table 2. During the review stage, reviewers can access it via a token-protected link, with detailed protocols provided in the Data availability section. Upon publication, the dataset will be publicly accessible on GitHub.

**Table 2:** Features of the SLID dataset.

Category	No. images with the label	No. images without the label
Pupil	2,303	314
Cornea	2,573	44
Conjunctiva	2,616	1
Cataract	225	2,392
Intraocular lens	119	2,498
Lens dislocation	40	2,577
Keratitis	222	2,395
Corneal scarring	69	2,548
Corneal dystrophy	300	2,317
Corneal/conjunctival tumor	488	2,129
Pinguecula	405	2,212
Pterygium	163	2,454
Subconjunctival hemorrhage	181	2,436
Conjunctival injection	307	2,310
Conjunctival cyst	113	2,504
Pigmented nevus	416	2,201

The original slit-lamp images are in PNG format and can be found in the “Original_Slit-lamp_Images” folder, named as “n.png”, and the respective annotated file is provided as “Annotations.csv”.

The “Original_Slit-lamp_Images” file contains 2,617 slit-lamp images, with the “n” ranging from 1 to 2,617 representing the respective image number. The dataset includes images at three resolutions: 2,576 × 1,934 pixels (1,412 images), 1,924 × 1,556 pixels (746 images), and 1,284 × 964 pixels (459 images). Of these 2,617 images, 245 are from patients with no detectable lesions, 2091 are from patients with a single lesion (monomorbidity), and 281 are from patients with multiple lesions (multimorbidity). For the multimorbidity subset, the mean number of lesions per image is 2.82, and the median is 3. It should be noted that keratitis is almost always accompanied by conjunctival injection; therefore, images with only these labels are included in the monomorbidity set.

The “Annotations.csv” contains five columns: “filename”, “file_size”, “annotation_count”, “annotation_ID”, “attributes”, and “shape_coordinates”. “filename” indicates the image name, “file_size” indicates the image size, “annotation_count” specifies the number of annotations, “annotation_ID” represents the annotation ID, “attributes” details specific anatomical regions and lesions, and “shape_coordinates” describes their respective localization.

## 3 Data validation and utility

### 3.1 Data validation

**Figure 2:**
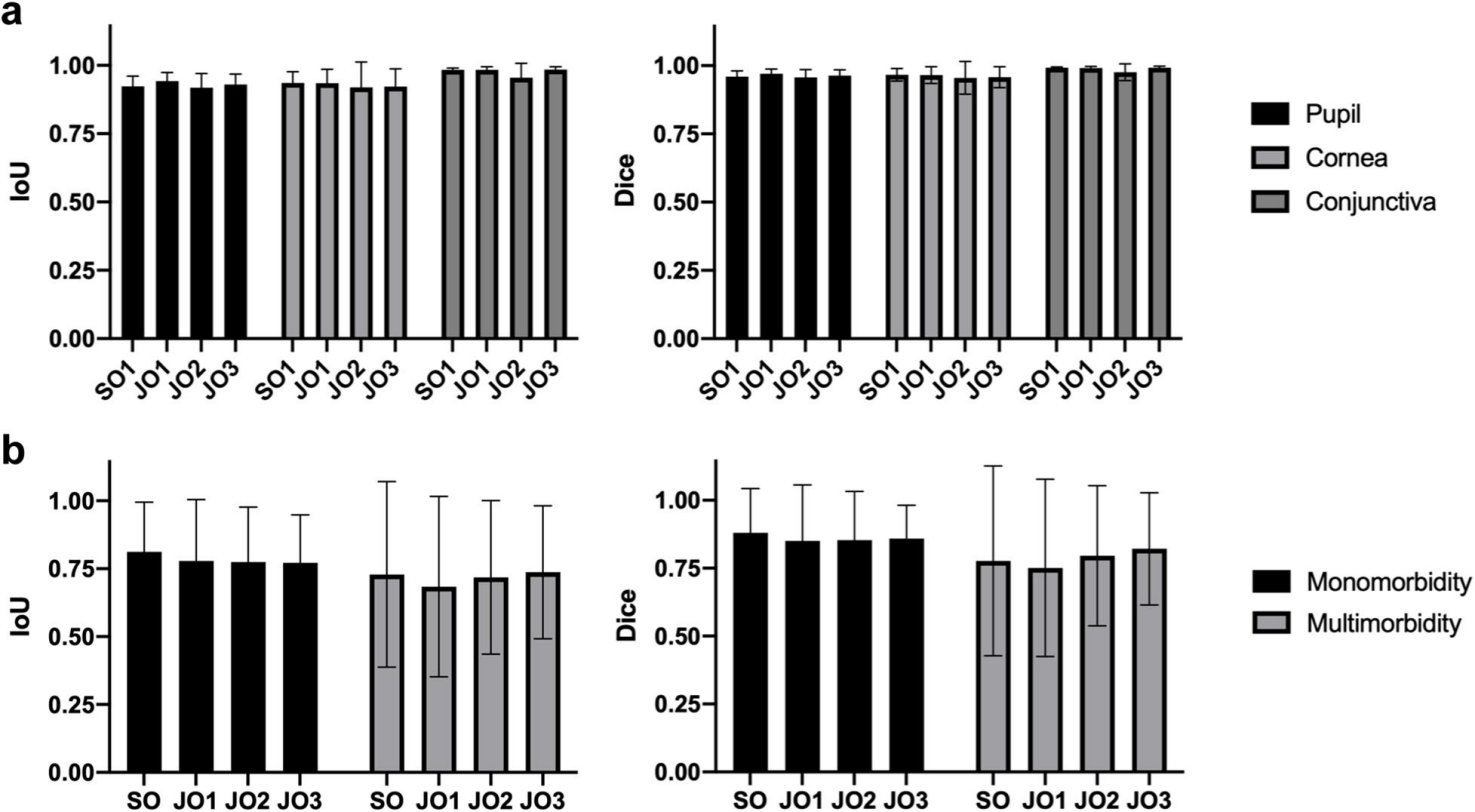
Validation of the SLID dataset. **(a)** The IoU and Dice score of the anatomic segmentation validation. **(b)** The IoU and Dice score of the lesion identification validation.

To validate the anatomic and lesion annotations, another one SO and three JOs were invited to anatomically segment 20 images and identify lesions in 195 images containing both monomorbidity and multimorbidity conditions randomly selected from the dataset. The results of anatomic segmentation and lesion identification are presented in Figure 2. All anatomic regions demonstrated excellent consistency, with mean IoU (Intersection over Union) >0.918 and mean Dice > 0.955 across all three regions and all four experts. Notably, segmentation performance for the conjunctival region was slightly superior to that of the corneal and pupillary regions, likely due to the clearer boundary between the conjunctiva and surrounding eyelid skin tissue. The validation results of lesion identification showed acceptable consistency, with mean IoU > 0.764 and mean Dice > 0.835 across all four experts. Among these images, those with monomorbidity exhibited relatively higher consistency, achieving a mean IoU of 0.785 and mean Dice score of 0.861. Images with multimorbidity also showed acceptable consistency, with a mean IoU of 0.717 and mean Dice score of 0.786. When analyzing specific lesion types, pterygium and pinguecula demonstrated lower consistency, primarily due to their less distinct lesion boundaries.

### 3.2 Data utility

To evaluate the potential of the proposed dataset for deep learning-based anterior eye multi-lesion detection, a YOLOv8 model was trained using single-lesion images following standard procedures, with experiment parameters listed in Supplementary Table S1 (25). At the image level, the dataset was randomly divided into training, validation, and test sets in an 8:1:1 ratio, achieving an average mean Average Precision (mAP) of 0.873 across all 13 single-lesion categories (Supplementary Table S2). At the patient level, we re-executed the 8:1:1 split, ensuring that all images from the same patient were assigned to a single dataset (training, validation, or test). Under this patient-level split, YOLOv8 achieved an average mAP of 0.736.

## Data Availability

De-identified data were used in the present study. These data are publicly accessible on GitHub at https://github.com/xumingyu-hub/SLID.
