# Supplementary material for: SLID: a slit-lamp image dataset for deep learning-based anterior eye anatomical segmentation and multi-lesion detection
Source: Front Digit Health. 2026 Jan 12;7:1716501. doi: 10.3389/fdgth.2025.1716501 (PMC12832839; doi:10.3389/fdgth.2025.1716501)
Supplement: Supplementary file 1 [file Datasheet1.pdf]

Supplementary Material

Table S1. The experimental parameter settings of the YOLOv8 model.

Parameters	Setting
initial learning rate	0.01
final learning rate	0.01
momentum	0.937
weight-decay	0.0005
optimizer	AdamW
batch size	16
epochs	300
imgsz	640
box	7.5
cls	0.5
dfl	1.5

Table S2. Performance of the YOLOv8 model across disease categories.

**Category**	**mAP**
Cataract	0.956
Intraocular lens	0.995
Lens dislocation	0.995
Keratitis	0.811
Corneal scarring	0.995
Corneal dystrophy	0.939
Corneal / conjunctival tumor	0.880
Pinguecula	0.664
Pterygium	0.879
Subconjunctival hemorrhage	0.741
Conjunctival injection	0.995
Conjunctival cyst	0.623
Pigmented nevus	0.871

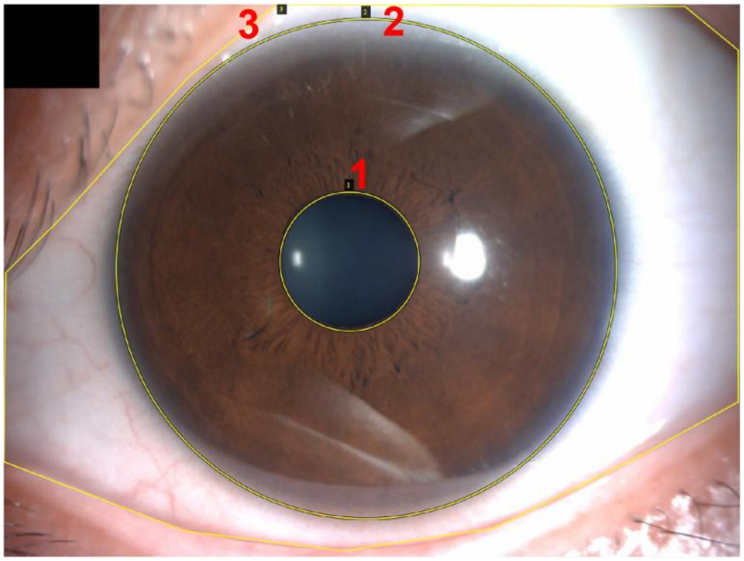

Figure S1. Example of anatomic annotation. (1) Pupil. (2) Cornea. (3) Conjunctiva.

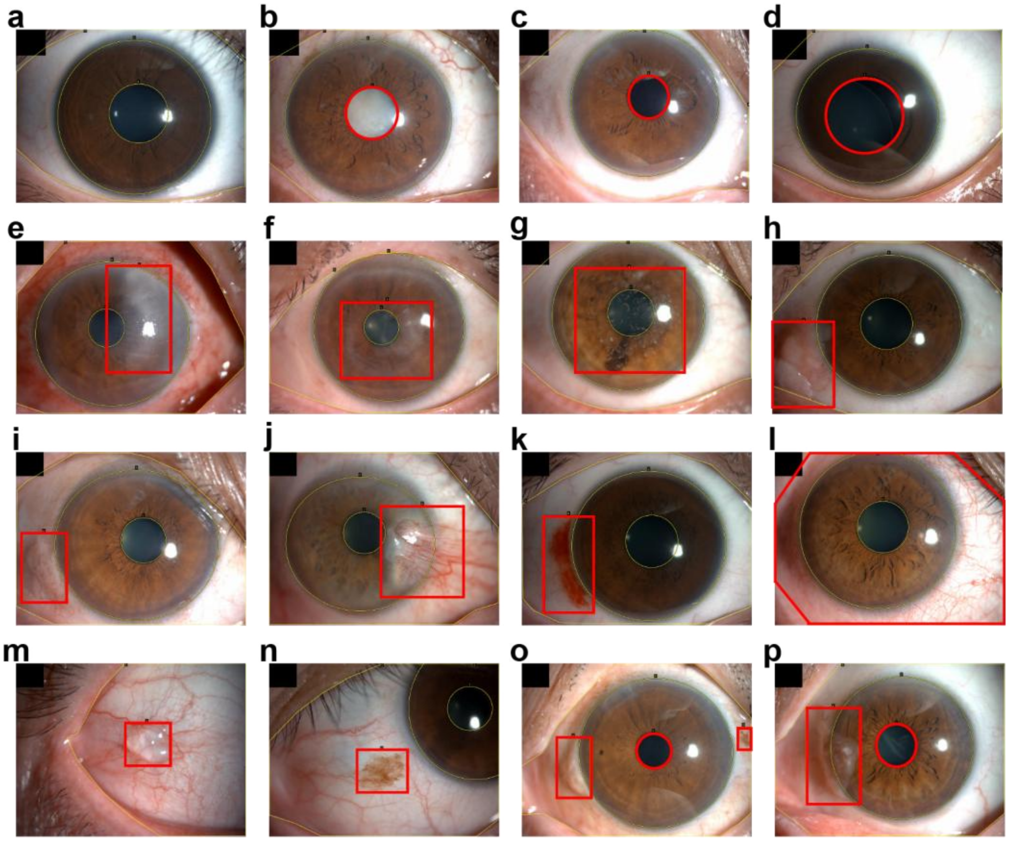

Figure S2. Examples of lesion identification, with lesions highlighted in red. (a) normal images, images with (b) cataract, (c) intraocular lens, (d) lens dislocation, (e) keratitis, (f) corneal scarring, (g) corneal dystrophy, (h) corneal/conjunctival tumor, (i) pinguecula, (j) pterygium, (k) subconjunctival hemorrhage, (l) conjunctival injection, (m) conjunctival cyst, (n) pigmented nevus, (o) images with multimorbidity of intraocular lens, pinguecula, and pigmented nevus, and (p) images with multimorbidity of cataract and pterygium.
